# Could Cognitive Behavioural Therapy Be an Effective Treatment for Long COVID and Post COVID-19 Fatigue Syndrome? Lessons from the Qure Study for Q-Fever Fatigue Syndrome

**DOI:** 10.3390/healthcare8040552

**Published:** 2020-12-11

**Authors:** Mark Vink, Alexandra Vink-Niese

**Affiliations:** 1 Family and Insurance Physician, 1096 HZ Amsterdam, The Netherlands; 2 Independent Researcher, 49032 Osnabrück, Germany; vinkniese.alexandra@gmail.com

**Keywords:** actometer, CBT, chronic fatigue syndrome, cognitive behavioural therapy, disability, long COVID, myalgic encephalomyelitis/chronic fatigue syndrome, ME/CFS, post-COVID-19 fatigue syndrome

## Abstract

An increasing number of young and previously fit and healthy people who did not require hospitalisation continue to have symptoms months after mild cases of COVID-19. Rehabilitation clinics are already offering cognitive behavioural therapy (CBT) as an effective treatment for long COVID and post-COVID-19 fatigue syndrome based on the claims that it is effective for myalgic encephalomyelitis/chronic fatigue syndrome (ME/CFS)—the most common post-infectious syndrome—as no study into the efficacy of CBT for post-COVID-19 fatigue syndrome has been published. Re-analyses of these studies, however, showed that CBT did not lead to objective improvements in heterogeneous groups of ME/CFS patients, nor did it restore the ability to work. The group of patients with long COVID and post-COVID-19 fatigue syndrome, on the other hand, is homogeneous. We therefore analysed the Dutch Qure study, as it studied the efficacy of CBT in a homogeneous group of patients who developed Q-fever fatigue syndrome—which affects up to 30% of patients—after the largest reported outbreak of Q-fever, to see if CBT might potentially be an effective treatment for long-haulers after COVID-19 infection. Our reanalysis found that the Qure study suffered from many serious methodological problems, which included relying on one subjective primary outcome in a study without a control group for the non-blinded CBT treatment group, using a post hoc definition of improvement, waiting 2 years before publishing their objective actometer results and ignoring the null effect of said results. Moreover, only 10% of participants achieved a clinically meaningful subjective improvement in fatigue as a result of CBT according to the study’s own figures. Consequently, CBT has no subjective clinically meaningful effect in nine out of every ten patients that are treated with it. Additionally, the subjective improvement in fatigue was not matched by an improvement in disability, even though the disability was fatigue related according to the researchers. On top of this, CBT did not lead to an objective improvement in physical performance. Therefore, it cannot be said that CBT is an effective treatment for Q-fever fatigue syndrome either. It seems therefore unlikely that CBT will reduce disability or lead to objective improvement in long COVID or in post-COVID-19 fatigue syndrome.

## 1. Introduction

The novel coronavirus disease 2019 (COVID-19), an infectious disease caused by the severe acute respiratory syndrome coronavirus 2 (SARS-CoV-2), has spread rapidly around the world [1]. Globally, as of 14 October 2020, there have been 38,002,699 confirmed cases, including 1,083,234 deaths, according to the World Health Organisation (WHO) [2]. An increasing number of young and previously fit and healthy people who did not require hospitalisation, continue to have symptoms months after mild cases of COVID-19 [1,3,4]. Commonly reported symptoms are severe and debilitating fatigue, breathlessness, headaches, muscle and/or joint pain, “brain fog,” memory loss, sensation of pressure on the chest, palpitations, nausea, dramatic mood swings in combination with exercise intolerance and a relapsing-remitting pattern of recurrence [1,3,4,5]. This is not surprising, as many of these symptoms have been widely observed after a variety of other viral and non-viral infections [6]. According to Dr. Anthony Fauci, the director of the US’s National Institute of Allergy and Infectious Diseases, and one of the most prominent international pandemic COVID-19 advisors, “It’s extraordinary how many people have a post-viral syndrome that’s very strikingly similar to myalgic encephalomyelitis/chronic fatigue syndrome” [7].

Professor Garner, the co-ordinating editor of the Cochrane Infectious Diseases Group, who was part of the original team that set up the Cochrane Collaboration, went to his occupational health services to get some help for his long COVID, and he was told that he “needed a course of cognitive behaviour therapy” (CBT) [8]. Others, such as American professor Hannah Davis and British professor Alwan, an epidemiologist and a public health consultant, have shared similar experiences [9,10]. Dr Faas, the former chairman of the Dutch association for insurance doctors, noted that long COVID patients are being told that they need CBT as for more than 20 years, doctors have been telling that to patients with other post-infectious diseases [11]. Additionally, for example, the Oxford Health NHS Foundation Trust and the British Royal Society of Medicine are already promoting CBT, not to help people cope with the debilitating post-COVID-19 problems, but as an effective treatment for post-COVID-19 fatigue syndrome [12,13,14,15]. Li et al. recently concluded that CBT is effective in reducing depression, anxiety and stress in hospitalised patients with COVID-19 when compared to no treatment (n = 93) [16]. However, no studies into the efficacy of CBT for post-COVID-19 fatigue syndrome have been published to date.

The assumption that CBT will be effective is based on CBT studies for myalgic encephalomyelitis/chronic fatigue syndrome (ME/CFS). However, the participant samples in those studies are very heterogeneous, not only because in only around 70% of participants the disease was triggered by an infection, but also because participants developed it after many different infections [6]. Additionally, a number of re-analyses found that in those studies, CBT did not lead to objective improvement, nor did it restore the ability to work [17,18]. However, in the case of post-COVID-19 fatigue syndrome and long COVID, the sample is not heterogeneous but homogeneous. This leads to a question: has a CBT study been undertaken for a homogeneous sample of patients with a post-infectious syndrome? The answer to that question is yes. Such a study was done in the Netherlands [19] after the largest reported Q-fever outbreak which occurred during 2007–2010 and was linked to dairy goat farms near densely populated areas in the southeast of the country. Q-fever, caused by the intracellular coccobacillus *Coxiella burnetii*, is a zoonosis (a disease caused by germs that are transmitted from animals to humans) that occurs worldwide [20,21]. It is presumed to have involved human exposure via a wind-borne route [21] and resulted in 4223 notifications of Q-fever [22]. Just like with COVID-19, a large proportion of patients (60%) remain asymptomatic. It is estimated that around 44,000 people contracted Q-fever during that outbreak, of which at least 95 people died [23]. Following acute Q-fever, up to 30% of patients develop Q-fever fatigue syndrome (QFS) which may last for ten years or more [24]. This is similar to the 27% who were found to have developed ME/CFS after surviving SARS four years earlier [25]. Chronic Q-fever needs to be excluded—IgG-phase I titre < 1:1024 in the last 3 months—before the diagnosis of QFS can be made [26]. This is of particular importance because untreated chronic Q-fever can be fatal [19]. 

The majority of patients with post Q-fever fatigue syndrome were also previously healthy persons with no underlying medical or psychological problems who developed a complex of symptoms dominated by debilitating fatigue after acute and symptomatic Q-fever infection [21]. Typical features of QFS include profound fatigue, arthralgia, myalgia, concentration and memory problems, sleeping problems, sweats and headaches [27]. QFS patients experience impaired health status, impairment of general and social functioning and a reduction in quality of life [24]. In many cases, QFS leads to long-term sick leave and substantial economic costs to patients, their families and society [24]. 

According to the Dutch QFS guidelines, CBT is the only effective treatment for QFS and the fatigue caused by it [26]. This conclusion is based on the Dutch Qure study [19,28,29]. In this article, we review the Qure study to see what lessons can be learnt from this study in relation to the efficacy of CBT for a post-infectious syndrome caused by one known infectious agent, by answering the following questions:

1. Does CBT lead to a clinically significant objective improvement in QFS?
2. Does CBT lead to a clinically significant improvement in the level of disability in QFS?
3. Does CBT restore the ability to work in QFS?

## 2. Summary of the Qure Study

The Qure study (Qure: Q-fever fatigue syndrome—response to treatment) was, according to the study itself, “the first randomized placebo-controlled trial” [19] “to assess the efficacy of long-term treatment with either doxycycline or CBT in patients with QFS” [30]. The study involved 155 adult patients with QFS. The study concluded that CBT is effective at treating fatigue in QFS, in comparison to a placebo or doxycycline, but that this effect had disappeared by the 1-year follow-up due to a decrease in self-efficacy, and therefore patients should be treated with booster sessions of CBT [29]. 

Patients in the medication arm of the study were treated with doxycycline 200 mg or placebo, both orally administered once daily for 24 weeks. Study visits for medical check-ups were at 4, 8, 16 and 26 weeks after the start of treatment. Patients allocated to CBT received approximately 24 weeks of individual CBT based on the manual of CBT for chronic fatigue syndrome (CFS) by trained and supervised CBT therapists. Treatment frequency was determined on an individual basis with sessions intended for once every two weeks. The primary outcome was fatigue on the checklist of individual strength (CIS) sub-scale of fatigue severity at 26 weeks (end of treatment) [19,29]. The CIS fatigue score indicates the level of fatigue experienced over the past 2-weeks. The score on this eight-item scale ranges from 8 (no fatigue) to 56 (maximally fatigued). Severe fatigue is defined by the literature as a score of 35 or more, and according to a study by three of the Qure authors, healthy adults with a mean age of 37.1 (SD 11.5)—mean age in the Qure study was 43.8 [28]—have a mean CIS fatigue score of 17.3 (SD 10.1) [31].

Secondary outcomes were level of functional impairment on the sickness impact profile (SIP8) with a cut-off score of 450 or more indicating significant disabilities, psychological distress, *Coxiella burnetii* serology and blood serum PCR [29].

### 2.1. Inclusion Criteria

QFS was defined as severe fatigue (CIS score ≥35) for ≥6 months, causing significant disabilities (SIP8 score ≥ 450) in daily functioning, not being caused by chronic Q-fever or other somatic or psychiatric morbidity, directly related to an acute Q-fever infection; and the fatigue should have been either absent before or have significantly increased since the acute Q-fever infection. Chronic Q-fever was excluded based on negative serum polymerase chain reaction (PCR), Q-fever serology (immunoglobulin G phase I titers < 1:1024), and absence of signs of endocarditis or vascular infection [19].

### 2.2. Rationale for Using CBT in QFS

The rationale for using CBT in Q-fever fatigue syndrome, according to the authors in their original publication from 2017, is the following [19]. “Cognitive-behavioral therapy (CBT), aimed at fatigue-related cognitions and behavior thought to perpetuate symptoms, can reduce symptoms and improve functioning in CFS. A considerable overlap in fatigue-perpetuating factors between QFS and CFS implies that CBT might also reduce fatigue severity in QFS.” However, in the same year in their reply to a comment by professor Raoult, they stated that, “We have found that the perpetuating factors in QFS patients clearly differ from those with chronic fatigue syndrome (CFS)” [32]. Interestingly enough, both statements reference back to the same article from 2015 [33], and five of the authors of that article also wrote the two contradictory statements in 2017. 

According to the Qure study, CBT for QFS “is a complex intervention” [19] that is “aimed at changing the beliefs and behaviors assumed to maintain fatigue” [30]. “CBT will consist of a protocolized intervention of 12 sessions during a period of 24 weeks” [34] and “is individually delivered by trained cognitive-behavioral therapists...according to a written treatment manual” [30]. It starts with goal setting and psycho-education on the possible roles of cognition and behaviour in maintaining the fatigue [34]. According to the Qure study’s protocol, “Goals usually include the resumption of work, hobbies, and other activities that imply that the patient is no longer severely fatigued and disabled, which is the goal of CBT for QFS” [30]. “The maintaining factors will subsequently be addressed (regulation of the sleep-wake cycle, gradual increasing activity, reformulating fatigue related cognitions)” [34]. 

Part of CBT for QFS is an activity program. This “activity program consists of daily walking or cycling, which is gradually increased. The increase in activity is not determined by the level of symptoms, but is time contingent. When patients succeed in increasing their physical activity, they also start to increase their social and mental activities. In the last phase of therapy, patients work systematically towards reaching their goals, which are formulated at the start of the therapy. Following this, they are encouraged to perceive feelings of fatigue as a normal part of an active and healthy life” [30]. Unfortunately, no objective evidence was presented that patients actually suffer from dysfunctional fatigue-related beliefs and that their symptoms are not caused by an underlying physical illness. Maybe because objective evidence to support this assumption does not exist.

### 2.3. Issues with the Protocol

The trial was registered with the clinical trials registry on 18 March 2011 as a randomised controlled trial. Its objective was “to assess the efficacy of two treatment strategies for fatigue and disabilities in QFS: long-term treatment with doxycycline or cognitive behavioral therapy (CBT)” [34]. Participants were recruited between April 2011 and September 2015 [34], yet the trial’s protocol was not submitted until 11 March 2013 and published on 27 March 2013 [30]. However, as Evans noted in his article on changing endpoints after the start of a clinical trial, “A fundamental principle in the design of randomized trials involves setting out in advance the endpoints that will be assessed in the trial, as failure to prespecify endpoints can introduce bias into a trial and creates opportunities for manipulation” [35]. 

The trial relied on one self-reported primary outcome—fatigue, as measured by a questionnaire [30]. An objective measurement of activity or work status was not used as a primary outcome according to the protocol, even though the authors noted when they registered their study that, “QFS leads to substantial morbidity and has a high socioeconomic burden related to increased use of healthcare facilities and absence from work” [34]. It is unclear why the study then did not use work status as a primary (or secondary) outcome, nor is it clear why disability was a secondary and not a primary outcome, if the objective of the study was to assess the efficacy in relation to fatigue and disabilities.

### 2.4. Issues with the Trial Design

The study was a randomised trial with two arms. Patients in the study were randomly assigned to the medication arm or to the CBT arm. In the medication arm a second randomisation was performed allocating patients to the doxycycline or the placebo group. There was no control group in the CBT arm of the study; therefore, the study compared results to the placebo group. Patients in the CBT group were seen 12 times—two-weekly over 24 weeks—even though patients in the other two groups were only “monitored [at] 4, 8, 16 and 26 weeks after start for side effects (rash, liver enzymes)” [34,36]. The authors acknowledged the issues with the design of their study by acknowledging that the CBT group was non-blinded by stating the following: “CBT was directly compared to placebo plus usual care [they could see their own doctor if needed], which might explain some of the differences observed as patients in the CBT group clearly know they are being treated.” They also stated though, that, “Due to the maximum number of available patients, it was not possible to include a control group [for the CBT group] without any form of treatment” [19]. However, a fundamental principle of a randomised controlled trial is “that the comparison/control group offers the same frequency and intensity of contact, positive expectations, attention and support” as the intervention group [37]. Otherwise, any difference in efficacy of the treatments between the two groups might be simply down to a poorly designed or absent control group. Interestingly enough, three of the authors had written the following in a CFS study. “In the absence of a control treatment group, it is difficult to attribute this effect to treatment with certainty” [31]. Why they did not write something similar in the Qure study is unclear. 

The chances of finding improvements after CBT, irrespective of whether it would lead to any benefits for the patient or not, were further increased by relying on a subjective instead of an objective primary outcome, alone or in combination with a subjective one. Consequently, if one does not know if the improvements in fatigue are caused by CBT or by the design of the study, then one cannot conclude that CBT is effective.

### 2.5. Issues with the Primary Outcome

One of the problems with assessing outcomes via questionnaires in a non-blinded trial of the form of CBT used in the Qure study is “response-shift bias.” This occurs when an intervention leads individuals to change their evaluations with regard to the dimension measured, leading the therapist (and often also the patient) to conclude erroneously that the treatment has worked [38]. This is even more of a problem when the therapy used, in this case CBT, aims to modify participants’ beliefs and perceptions of their symptoms.

Other important causes of bias are “effort justification,” where patients investing substantial time, energy and effort into an intervention often feel a psychological need to justify this commitment. There is also a tendency for patients/clients to report improvement in accordance with what they believe to be the therapist’s/researcher’s hypothesis [38].

Wood et al., in a large-scale meta-analysis of over 1300 varied clinical trials, found that in non-blinded trials, subjectively assessed outcomes increase the degree of bias, and that introducing objective outcomes reduces this [39]. Other systemic reviews of clinical trials also concluded that lack of patient blinding combined with self-reporting of outcomes leads to pronounced bias, as they become prone to outside influences, leading to the erroneous inference of efficacy in its absence, thereby making such trials unreliable [38,40]. One cannot safely conclude that CBT is an effective treatment, in view of the fact that the trial did not use an objective primary outcome to correct for a number of different biases. 

## 3. Results

### 3.1. Effect Size

According to the authors in their protocol, “a moderate controlled effect size of 0.53” was needed to demonstrate “a significant difference between the results in the treatment groups and in the placebo group” [30]. As can be seen in Table 1, neither CBT nor doxycycline achieved that. Moreover, a systematic review by Hróbjartsson et al. [40] concluded that there is pronounced bias due to lack of patient blinding in clinical trials with patient-reported outcomes and that "nonblinded patients exaggerated the effect size by an average of 0.56 standard deviation." Table 1 also shows that the effect size of CBT versus placebo, and doxycycline vs. placebo for fatigue, disability and psychological distress, are less than the effect size of relying on subjective outcomes in non-blinded studies, as found by Hróbjartsson et al.

### 3.2. Fatigue Severity Scores at Follow-Up

The authors reported at follow-up—52 weeks from baseline and 26 weeks after completion of the original trial—the following: “Fatigue severity in the CBT, but not in the doxycycline or placebo group was significantly increased at followup compared to EOT [end of treatment]. Fatigue severity scores of CBT (adjusted mean 39.8) and doxycycline (adjusted mean 41.0) groups did not significantly differ from the placebo group (adjusted mean 37.1; *p* = 0.92 and *p* = 0.38, respectively)” [28]. That can also be seen in Table 2; it also shows that patients in all three groups were on average still severely fatigued and still ill enough to re-enter the trial and receive the same treatment again.

### 3.3. Post Hoc Definition of Clinically Meaningful Improvement in Fatigue

Clinically meaningful improvement in fatigue was not defined in the protocol [30], nor when the trial was registered [34,36,41]. Instead, it was defined in the original publication after the study was over. Consequently, it was a post hoc definition. This is contrary to normal practice where endpoints are defined prior to trial commencement [35]. This means that the results may have influenced this definition. Additionally, this—as pointed out by Goldacre in an article entitled “how researchers dupe the public with a sneaky practice called ‘outcome switching’”—“allows the...‘random error’ in your data to exaggerate your results (or even yield an outright false positive, showing a treatment to be superior when in reality it’s not),” leading to the wrong conclusions, and “in medicine, that’s not a matter of academic sophistry—it causes avoidable suffering” [42]. Additionally, according to Stanford professor Ioannidis, “Flexibility increases the potential for transforming what would be “negative” results into “positive” results,” and “the greater the flexibility in designs, definitions, outcomes, and analytical modes...the less likely the research findings are to be true” [43]. The flexibility in a post hoc definition of improvement is high, and using a post hoc definition could enable authors to tailor fit this to the results.

According to the supplementary data of the original article, a clinically meaningful improvement was defined as a “CIS fatigue severity score of < 35...and...a minimal drop of nine points on the CIS subscale fatigue severity” [19]. This “was reached by 31% and 29% of patients in the doxycycline; 46% and 40% of patients in the placebo condition; and 56% and 24% of patients in the CBT group, showing a significant relapse in the latter (*p* = 0.01). While a significant difference between groups was seen at EOT (*p* = 0.03), this was no longer the case at followup (*p* = 0.18)” [28]. In other words, there was no significant difference in clinically meaningful improvement in fatigue at follow-up.

Table 3 shows the clinically meaningful improvement in fatigue at the end of treatment using the trial’s own definition for clinically meaningful improvement. This was achieved by 56% and 46% in the CBT group and placebo group, respectively. Consequently, only 10% of participants achieved a clinically meaningful improvement in fatigue at the end of treatment as a result of CBT. In other words, ten participants need to be treated with CBT for one to achieve clinically meaningful improvement in fatigue. One cannot safely conclude that a treatment, in this case CBT, is effective when it has no meaningful effect in nine out of every ten patients that are treated with it.

### 3.4. Functional Impairment

The SIP8 questionnaire measures functional disability in ambulation, home management, mobility, alertness behaviour, sleep/rest, work limitations, social interactions, recreation and pastimes. The mean SIP8 total score of healthy women, according to a study by three of the Qure authors, is 65.5 (SD 137.8) [31]. According to the Qure study itself, “A cut-off score of 450 or more” indicates “significant disabilities” [19]. This was also its entry requirement.

The study concluded that “no significant differences in mean functional impairment scores were found between EOT and follow-up assessment in the CBT, doxycycline or placebo group,” as can also be seen in Table 4. However, it also concluded that, “No significant differences in functional impairment at follow-up were found between the CBT and placebo group, or doxycycline and placebo group” [28]. Table 5 shows that there were also no statistically significant differences in treatment effect on functional impairment between the three groups at the end of treatment.

Consequently, none of the treatments were effective for functional impairment, and patients in all three groups were on average not only still severely disabled, but also still disabled enough to re-enter the study and be treated with the same treatment again. In view of that, one cannot safely conclude that CBT is an effective treatment.

### 3.5. Drop Outs

Empirical evidence suggests that participants who adhere to treatment tend to do better than those who drop out [44]. A systematic review by Abraha et al. [45] into the way systematic reviews report, found that if more than 10% of randomised patients have missing outcomes, then this will expose the trials to bias and/or it will reduce their power and precision. The percentages of participants in each trial arm that dropped out of the Qure study are the following:

- 15% (8/51), CBT;
- 6% (3/52), doxycycline;
- 4% (2/52), placebo [19].

The difference in drop out rate between the CBT and placebo group is substantial. Therefore, selective drop out may be an issue. Consequently, this might have exposed the results of the CBT group to bias, leading to further doubt about the efficacy of CBT.

### 3.6. Assessing Physical Activity

According to the protocol, the study was to measure physical activity/performance objectively by using the actometer at baseline and at the end of treatment, and subjectively by using the Physical Activity Rating Scale (PARS) [30]. The mediation analysis noted that, “The actometer is a reliable and valid instrument for the assessment of physical activity” [29] which was “worn day and night during a period of twelve consecutive days” [30]. However, neither the actometer results nor the PARS scores were published when the original Qure study was published in 2017 [19] and the reason for this was not given. Heneghan et al. refer to this type of reporting as a typical example of “outcome reporting bias,” which “occurs when a study has been published, but some of the outcomes measured and analysed have not been reported,” which “significantly affects the validity” of a study [46].

The actometer results, and the results of the Physical Activity Rating Scale, were published in a table in the mediation article [29], two years after they should have been published in the original article. However, they were not discussed. As can be seen in Table 6, CBT does not lead to objective or subjective improvement of physical activity.

A rheumatoid arthritis study by Rongen-van Dartel et al., which included two of the Qure researchers, concluded that there is an inverse relationship between fatigue and physical functioning/activity [47]. This suggests that the small subjective improvement of fatigue in a small number of patients after CBT over a placebo was simply an artefact.

## 4. Discussion

2020 is the year of the COVID-19 pandemic, and so far millions of people have been infected. Many of them still have symptoms long after the initial infection disappeared. At the moment, it is unclear how many of them will recover spontaneously over time, how many have organ damage and how many patients are developing post-infectious illnesses. Rehabilitation clinics are offering CBT for these patients based on the claims that these therapies are effective for ME/CFS—a post-infectious illness which can develop after many different infections—as no study into the efficacy of CBT for post-COVID-19 fatigue syndrome has been published. Over the last couple of years, a number of re-analyses of these ME/CFS studies have been published which show that CBT does not lead to objective improvement in heterogeneous groups of ME/CFS patients with different infections triggering their ME/CFS, nor does it restore the ability to work [17,18]. The group of patients with post-COVID-19 fatigue syndrome, on the other hand, is homogeneous. We therefore analysed the Dutch Qure study, as it studied the efficacy of CBT in a homogeneous group of patients with Q-fever fatigue syndrome, which affects up to 30% of patients after a symptomatic infection with Q-fever, to see whether CBT reduces disability, restores the ability to work and leads to objective improvement in QFS. By doing so, this reanalysis provides insights into the question of whether CBT might potentially be an effective treatment for long-haulers after COVID-19 infection.

### 4.1. The Qure Study

The Qure study was a randomised trial that compared the effectiveness of CBT with that of doxycycline and placebo. It used a questionnaire to assess its primary outcome (fatigue). The trial concluded in 2017 that, “CBT is effective in reducing fatigue severity in QFS patients. Long-term treatment with doxycycline does not reduce fatigue severity in QFS patients compared to placebo” [19]. In their follow-up article, they concluded that, “The beneficial effect of CBT on fatigue severity at EOT [end of treatment] was not maintained 1 year thereafter. Due to its initial beneficial effect and side effects of long-term doxycycline use, we still recommend CBT as treatment for QFS” [28].

Additionally, in 2019 they published a mediation analysis [29] in which they concluded that, “The decrease in fatigue brought on by CBT was completely mediated by an increase in self-efficacy with respect to fatigue. A reduction in self-efficacy partly mediated the increase in fatigue at followup in the CBT group.” And according to the study, patients should therefore receive “booster sessions focusing on restoration and maintenance of self-efficacy with respect to fatigue,” as that “may lead to elongation of the initial positive effects of CBT for QFS.”

According to the clinical trials registration in 2011, "the objective of the study is to assess the efficacy of two treatment strategies for fatigue and disabilities in QFS: long-term treatment with doxycycline or cognitive behavioural therapy (CBT)" [34]. It is unclear why the trial then chose to rely on one primary outcome (fatigue) instead of using disability as a primary outcome as well.

### 4.2. Non-Blinded Interventions

Trials of behavioural interventions are non-blinded by definition, and a number of recent articles have highlighted the problems of erroneous inferences of improvement in their absence, if non-blinded trials rely on subjective outcomes. To prevent that, those studies should use objective primary outcomes (as well) [38,40,48]. The Qure study could have easily done that, if they had used the objective actometer as a primary outcome, as they were already using it. The authors noted that, “QFS leads to substantial morbidity and has a high socio-economic burden, related to increased use of healthcare facilities and absence from work” [34]. According to their own protocol, “Goals usually include the resumption of work, hobbies, and other activities that imply that the patient is no longer severely fatigued and disabled, which is the goal of CBT for QFS” [30]. Why the Qure study then did not use work status as a primary (or secondary) outcome is unclear—especially as for employers, society and especially patients, that would be (one of) the most important and relevant outcome(s). Moreover, an influential systematic review by Whiting et al. of interventions for the treatment and management of chronic fatigue syndrome from 2001—three of the Qure researchers have been leading CFS researchers in the Netherlands since the 1990s—also concluded that subjective outcomes may be unreliable because “persons may feel better able to cope with daily activities because they have reduced their expectations of what they should achieve, rather than because they have made any recovery as a result of the intervention. A more objective measure of the effect of any intervention would be whether participants have increased their working hours, returned to work...or increased their physical activities” [49].

Furthermore, the BRANDO project (Bias in Randomised and Observational studies) [48], which amongst others included Stanford professor Ioannidis, concluded in 2012 that “as far as possible, clinical and policy decisions should not be based on trials in which blinding is not feasible and outcome measures are subjectively assessed” because lack of blinding is “associated with an average 13% exaggeration of intervention effects […] Therefore, trials in which blinding is not feasible should focus as far as possible on objectively measured outcomes.” The Qure study unfortunately failed to do this.

The authors acknowledged that a limitation of the study “is that patients in the CBT group were non-blinded as masking for CBT is not possible.” Additionally, that they should have used “another comparison arm than placebo, e.g., waiting-list,” but that that “was not optional” because of “the major burden for QFS patients and the limited number of eligible patients at the time” [29]. However, when the trial started, there were more than 4200 cases in which the authorities had been notified of Q-fever [22]. According to the authors themselves, at least 20% of cases will develop Q-fever fatigue syndrome. A systematic review by Morroy et al. from 2016 [24], which included three Qure study authors, found that “Q-fever fatigue syndrome...has been described worldwide in up to...30% of patients.” Consequently, there would have been at least 800 and maximally about 1200 QFS patients, concentrated in the south east of the Netherlands. And because of the “major burden,” it would seem more logical that patients would want to take part in the first treatment study in the Netherlands for Q-fever fatigue syndrome, for which there is no effective treatment, than in epidemiological studies.

The authors continued by saying, “We therefore chose to compare CBT to placebo plus usual care. By comparing the additional effect of CBT to the placebo effect of receiving treatment, our mediation model assesses the specific contribution of CBT to the reduction of fatigue” [29]. However, they compared a non-blinded CBT group in which patients had 12 sessions of an hour to a group where patients had only four check ups—to see if they had developed a rash and to take blood samples for doxycycline levels—instead of 12 similar sessions, like the CBT group of, for example, relaxation. On top of that, they used a subjective primary outcome. Lack of patient blinding combined with self-reporting of outcomes leads to pronounced bias, as patients become prone to outside influences, leading to the erroneous inference of efficacy in its absence, thereby making subjectively assessed outcomes unreliable [38,40,50]. The Qure study itself noted in its original publication [19] that “CBT was directly compared to placebo plus usual care, which might explain some of the differences observed as patients in the CBT group clearly know they are being treated.” Moreover, response shift bias, which is an important problem due to the nature of the form of CBT used in this study, as discussed earlier, might be another reason for those differences. The only way to correct for that in a non-blinded study would be by using an objective primary outcome. Consequently, by relying on a patient-reported outcome in a non-blinded study, the study did not assess the specific contribution of CBT to the reduction of fatigue.

### 4.3. The Regression to the Mean Effect

Many patients enrol in clinical trials when their symptoms have flared up, yet with time these symptoms normally become less severe simply by random fluctuations and natural improvement, even when no treatment is used. This statistical phenomenon is called regression to the mean (RTM). If RTM is not fully controlled for, then it will lead to the erroneous conclusion that the improvement was down to the treatment, “even if the treatment has no effectiveness whatsoever” [51].

In medicine, randomisation together with a placebo control group are used to remove the effect of RTM, which according to general consensus is the best approach to control for it [51,52,53]. It is impossible to use placebo control groups in studies of psychological treatments. Therefore, in psychology, patients in the control group need to be offered a control treatment, for example, relaxation. This way, scores in both groups should be equally affected by RTM, and the difference in mean change between the two groups should be attributable solely to the effects of the treatment under investigation. A study without a control group, or with a waiting list or a no-treatment control group, often called usual care, does not remove the effect of RTM [51,52].

Additionally, baseline scores should be based on the mean of multiple tests rather than just a single one. This has the effect of stabilizing the mean and reducing within-subject variability [54].

The Qure study did not have a control group for the CBT treatment arm, nor did it stabilise its mean baseline scores. Consequently, the study did not correct for regression to the mean, and therefore the subjective improvement in fatigue might well have been caused by regression to the mean and not by the treatment under investigation.

### 4.4. Self-Efficacy

According to the study, “A decrease in self-efficacy with respect to fatigue was found to be the only mediator of the relapse of fatigue after completion of treatment.” And they recommended booster sessions, because according to them, without “active support from the therapist, patients seem to fall back in their old beliefs of not having control over their fatigue” [29]. In other words, patients are blamed for the decrease in efficacy of CBT. However, as noted earlier, a systematic review by Hróbjartsson et al. [40] concluded that non-blinded patients exaggerated the effect size in clinical trials with patient-reported outcomes by an average of 0.56 standard deviation. According to the Qure study, the effect size of CBT on fatigue at the end of treatment was 0.49 when compared to placebo. This is less than the effect size of relying on subjective outcomes in non-blinded studies, as found by Hróbjartsson et al. It is also almost 10% less than the effect size of 0.53 which, according to the Qure study’s protocol, was needed for CBT to be moderately effective [30].

### 4.5. Problems with the Protocol and a Post Hoc Definition of Clinically Meaningful Improvement

The protocol was published 2 years into a 4-year long trial, even though the “pro” in protocol means that it should be published before the start of the study. According to an article on changing endpoints after the start of a clinical trial, and as discussed earlier, endpoints should be set out in advance, “as failure to pre-specify endpoints can introduce bias into a trial and creates opportunities for manipulation” [35].

Additionally, as discussed earlier, the study waited to define a clinically meaningful improvement in fatigue until the study was over, contrary to normal practice where endpoints are defined prior to trial commencement [35]. This means that the results may have influenced this definition. Why the study came up with a post hoc definition of clinically meaningful improvement is unclear.

Moreover, if we use the trial’s own definition for clinically meaningful improvement in fatigue—defined as a “CIS fatigue severity score of <35...and...a minimal drop of nine points on the CIS subscale fatigue severity” [19]—and we use the trial’s own figures, then at the end of treatment, clinically meaningful improvement in fatigue was achieved by 56% and 46% in the CBT group and the placebo group, respectively, as can be seen in Table 3. Consequently, only 10% of participants achieved clinically meaningful improvement in fatigue at the end of treatment as a result of CBT. This means that ten QFS patients need to be treated for one to achieve that. To put it differently, for nine out of ten patients, CBT would not have a meaningful effect. In view of that, one cannot conclude that CBT is effective for fatigue.

### 4.6. No Effect on Disability

According to the clinical trials registry, the objective of the study was “to assess the efficacy of two treatment strategies for fatigue and disabilities in QFS: long-term treatment with doxycycline or cognitive behavioural therapy (CBT)” [34]. The disability entry requirement for the study was an SIP8 score of 450 or more, which according to the study means “substantial fatigue-related disabilities” [19]. When the study concluded that CBT was effective for fatigue at the end of treatment, it did not highlight that CBT was not effective for disability and that patients remained severely disabled, as can be seen in Table 4. However, if the improvement in fatigue had been a real improvement, and not simply a subjective one, then a similar improvement in disability would have been expected, especially as disabilities in QFS are fatigue-related according to the study itself.

### 4.7. Not Publishing Objective Outcomes

According to the protocol of the Qure study, “Doxycycline levels will only be determined in participants receiving doxycycline, and results will be kept secret until the entire study is completed. After completion, it is known whether doxycycline levels were sufficient to sort out effect” [30]. However, the study did not publish these doxycycline levels, and without these it is impossible to know whether doxycycline levels were actually sufficient or not.

According to the Qure study’s investigators, CBT is an effective treatment for CFS and CBT was tried for Q-fever fatigue syndrome because there are many similarities with CFS. Three CFS trials which play an important role in this evidence-base had reported subjective improvements and declared CBT to be an effective treatment for CFS. However, just like the Qure study, they had failed to report their actometer results. Wiborg et al., who included authors of these three studies, reanalyzed these studies, and they found that CBT did not lead to objective improvements in chronic fatigue syndrome [55]. Two of the authors of Wiborg et al. were involved in the Qure study too. Noteworthy is that one of them was also involved in all three of these studies that had failed to report their actometer results when the results were initially reported. Another author was involved in one and a third author of the Qure study was involved in two of these three studies [56,57,58].

The Qure study did not publish its actometer results in its original publication. Instead, the results were published 2 years later in a table in the mediation analysis. However, these results were not discussed in the article and their null effect was ignored. Why the study chose to ignore their own actometer results is unclear, although pressure on the study to find a positive result must have been high. The reason for this is that the Dutch Q-fever fatigue syndrome guideline, which was created by a multidisciplinary committee, which included four of the authors of the Qure study [30] and was published in 2012 [59], had already recommended CBT for QFS. It did so because according to these guidelines, CBT for CFS is safe and effective and there are many similarities between CFS and QFS. The fact that no study into the effects of CBT for QFS had been undertaken and that the Qure study was 3 years from completion and 5 years from publication, was apparently not a problem. Paradoxically enough, the committee strongly recommended not to use antibiotics for QFS because there was not sufficient evidence for its efficacy in QFS. This recommendation by the Dutch guideline of CBT for QFS could have raised expectations and hope in up to three-quarters of the participants of the Dutch Qure study who were enrolled into the study after the publication of the guideline. Anything that increases expectations and hope in participants is, according to a study by Cuijpers and Cristea [60], one of several methods available to help researchers show that their therapy is effective, even when it is not. According to them, these methods also “include a strong allegiance towards the therapy...making use of the weak spots of randomised trials (risk of bias), small sample sizes and waiting list control groups,” or no control group, as was the case for the CBT arm of the Qure study. Additionally, according to the same study, “If all that fails one can always not publish the outcomes.” Many of these methods were seen in the Qure study. For example, three of its researchers are strong proponents of the “unhelpful cognitions” theory of ME/CFS, which they and other colleagues had originated and/or actively promoted. This same theory was also the basis for using CBT in the Qure study. If their study had failed to show significant improvement and recovery, this would have undermined the very theories of reversibility to which these three investigators have dedicated their careers. Consequently, the risk of latent bias was palpable from the outset [61]. Moreover, according to a systematic review by Dragioti et al., the “experimenter’s allegiance effect inflates the reported effect sizes in randomized controlled trials in psychotherapy by 30%” [62]. Further bias was introduced into the non-blinded Qure study by relying on one subjective primary outcome and by selective outcome publishing and reporting.

### 4.8. The Cognitive–Behavioural Model for QFS: Fact or Fiction?

“CBT is a complex intervention, encompassing a stepwise increase in physical activity and challenging dysfunctional fatigue-related beliefs” according to the study [19]. Part of CBT for QFS is an “activity program” which “consists of daily walking or cycling, which is gradually increased. The increase in activity is not determined by the level of symptoms, but is time contingent” [30]. In other words, patients were following an activity programme with gradually increasing levels of activity for 24 weeks, irrespective of their symptoms. One would therefore expect a significant increase in activity after 24 weeks of daily training if there was no underlying physical illness preventing such an increase, and if patients were newly suffering from dysfunctional beliefs as the causes of their symptoms and problems, as was the assumption of the Qure study. For comparison, patients with stable chronic heart failure improved their 6-minute walk test results by 65% after only three weeks of exercising [63]. The outcome of the actometer, however, shows that an activity program of 24 weeks of daily training in patients with Q-fever fatigue syndrome does not lead to objective improvement.

The fact that CBT also does not improve disability (as measured by the SIP-8) and that there was only a short-lived subjective effect on fatigue in just one in every ten patients treated—which was less than the effect expected of using a subjective outcome in a non-blinded trial—raises serious doubts about the cognitive–behavioural (CB) model and the assumption that patients with Q-fever fatigue syndrome suffer from “dysfunctional fatigue-related beliefs.” It also suggests that it may be inappropriate to use CBT to treat Q-fever fatigue syndrome.

### 4.9. Strengths and Weaknesses of the Qure Study

A particular strength of the trial was that they used the actometer, an objective measure of activity, to measure the efficacy of CBT objectively, and they also used 97.5% confidence intervals when reporting most estimated effects. Other strengths of the study were that it was a fairly large randomised trial, that chronic Q-fever was excluded and that they also did a one-year follow-up.

Some of its weaknesses were:

(1) A protocol that was published 2 years after the trial started;
(2) No control group for the non-blinded CBT arm of the study;
(3) The percentage of participants with a co-morbid depression and/or anxiety, for which CBT is the most effective treatment according to a meta-analysis [64], was unclear;
(4) Not taking into account that 22% (43/200) of eligible participants refused to take part because they did not want to be treated with CBT;
(5) Relying on a subjective primary outcome in a non-blinded study;
(6) Using end of treatment as the primary outcome point even though individuals often experience a decline in therapeutic benefit within weeks after completing CBT [38];
(7) Ignoring the substantial difference in drop-out rates between CBT and doxycycline/placebo;
(8) Combining the doxycycline and placebo group in the mediation analysis and labelling it the medication group which gives the impression that all patients in this group were treated with medication whereas half of the group were treated with a placebo instead;
(9) Not publishing their doxycycline levels;
(10) Not correcting for regression to the mean;
(11) Using a post hoc definition of clinically meaningful improvement;
(12) Labelling a treatment effective which has no meaningful effect in 90% of patients;
(13) Waiting 2 years to publish the objective actometer results;
(14) Subsequently ignoring the actometer’s null effect;
(15) Ignoring the null effect on subjective activity;
(16) Ignoring the null effect on disability.

## 5. Conclusions

Reanalysis of the Qure study shows that it suffered from a number of serious methodological problems, which included relying on one subjective primary outcome in a study without a control group for the non-blinded CBT treatment group; and waiting 2 years before publishing their objective actometer results, and then ignoring their null effect. It also showed that CBT, just like treatment with a placebo or doxycycline, does not lead to objective improvement of activity or improvement in self-reported disability. This confirms the outcome of the re-analyses of CBT studies for heterogeneous groups of ME/CFS patients. Therefore, it can be argued that it is unlikely that the effect of CBT for long COVID and post-COVID-19 fatigue syndrome will be any different.

## Figures and Tables

**Table 1:** The standardized effect size on fatigue, disability and psychological distress at the end of treatment.

Outcome Measure	Doxycycline vs. Placebo	Doxycycline vs. Placebo, *p*-Value	CBT vs. Placebo	CBT vs. Placebo, *p*-Value	Effect Size of Using Subjective Outcomes in a Non-Blinded Trial [40]
CIS subscale fatigue severity	0.24	0.45	0.49	0.03	0.56
SIP8 total score	0.20	0.51	0.26	0.30	0.56
SCL-90 total score	0.18	0.45	0.43	0.01	0.56

SCL-90, symptom checklist 90 was used to measure the level of psychological distress; SIP8, sickness impact profile was used to measure the level of functional impairment; vs., versus. Source: [19].

**Table 2:** Mean fatigue severity scores at follow-up.

Outcome Measure	CBT	Placebo	Doxycycline	Severe Fatigue	Fatigue Inclusion Score
Fatigue severity	39.5	37.1	41.1	35 or more	35 or more

Source: [28].

**Table 3:** Percentage of participants with a clinically meaningful improvement in fatigue at the end of treatment.

Outcome Measure	Doxycycline (*n* = 52)	Placebo (*n* = 52)	CBT (*n* = 52)	*p* Value *	CBT vs. Placebo
Clinically Meaningful Improvement	31% (16)	46% (24)	56% (28)	0.03	10%

* *p* values were based on the Chi-square test for comparison of the three groups [19]. Clinically meaningful improvement in fatigue was defined by the study as a “CIS fatigue severity score of <35...and...a minimal drop of nine points on the CIS subscale fatigue severity” [19].

**Table 4:** Mean functional impairment scores at end of treatment and follow-up.

Treatment	EOT	Follow Up	Change Score	*p* Value	Significant Disabilities [19].	Score of Healthy People [31].
CBT	811.8	880.6	68.8	0.40	450 or more	65.5
Doxycycline	1092.1	1057.9	−34.2	0.65	450 or more	65.5
Placebo	949.3	853.0	−96.2	0.19	450 or more	65.5

EOT: end of treatment; source: [28].

**Table 5:** Treatment effect on functional impairment.

Outcome Measure	Doxycycline vs. Placebo, Mean Difference	Doxycycline vs. Placebo, *p*-Value	CBT vs. Placebo, Mean Difference	CBT vs. Placebo, *p*-Value
Functional impairment at EOT	−137.7	0.51	177.0	0.30
Functional impairment at FU	−200.2	0.42	8.1	>0.99

EOT, end of treatment; FU, follow-up; vs., versus. Source: [28].

**Table 6:** Differences between CBT and medication groups in changes in physical activity level from baseline to the end of treatment.

Outcome Measure	Difference between CBT and Medication Group	*p*-Value CBT vs. Medication Group	Effect Size
Physical activity level measured via the actometer	0.77	0.75	0.04
Physical Activity Rating Scale (PARS)	2.62	0.24	0.18

Source: Medication group: the authors combined the doxycycline and placebo group and labelled it the medication group in the mediation analysis [29].
